# Bispecific antibody-drug conjugates in cancer: therapeutic-window determinants, tumour-immune interactions, and clinical translation

**DOI:** 10.3389/fimmu.2026.1974912

**Published:** 2026-09-18

**Authors:** Yang Li, Yu Li, Rong Huang, Zhi-qiang San, Jun-feng Ye

**Affiliations:** 1 General Surgery Centre, First Hospital of Jilin University, Changchun, Jilin, China; 2 School of Nursing, Jilin University, Changchun, Jilin, China; 3 Department of Ophthalmology, First Hospital of Jilin University, Changchun, Jilin, China

**Keywords:** bispecific antibody-drug conjugates, immune checkpoint blockade, immunogenic cell death, izalontamab brengitecan, therapeutic window, tumour-immune microenvironment

## Abstract

Antibody-drug conjugates (ADCs) have established targeted payload delivery as an important anticancer strategy, but their efficacy remains constrained by antigen heterogeneity, incomplete internalisation, resistance and dose-limiting toxicity. Bispecific antibody-drug conjugates (BsADCs) add a second antigen- or epitope-binding specificity with the aim of broadening tumour coverage or altering receptor trafficking. The field crossed a major translational threshold in 2026, when izalontamab brengitecan (iza-bren; BL-B01D1), an EGFR×HER3 BsADC, received its first regulatory approvals in China for previously treated recurrent or metastatic nasopharyngeal carcinoma and oesophageal squamous cell carcinoma, while phase III studies also showed survival benefit in triple-negative breast cancer. These developments shift the central question from whether bispecificity can produce antitumour activity to when it can generate a clinically meaningful therapeutic window. This review evaluates BsADC development through six linked determinants: target-pair biology and co-expression, binding geometry and avidity, internalisation and lysosomal trafficking, linker-payload chemistry and drug-to-antibody ratio, intratumoural distribution and bystander activity, and biomarker-guided dosing and patient selection. We further examine how payload-mediated immunogenic cell death, Fc/Fcγ receptor-dependent uptake, suppressive tumour-immune niches and checkpoint-based combinations may modify activity beyond target recognition. Clinical programmes illustrate divergent outcomes: iza-bren provides regulatory validation of the modality, whereas experience with MEDI4276, zanidatamab zovodotin and REGN5093-M114 shows that enhanced binding or internalisation does not guarantee sustainable development. Importantly, much of the mechanistic and combination evidence for immune activation still derives from monospecific ADCs; immune benefit should therefore not be attributed to bispecificity itself without direct evidence. Future progress should integrate human pharmacokinetic/pharmacodynamic data, target-pair and immune-contexture biomarkers, dose-exposure-toxicity relationships and comparative value over optimized monospecific ADCs.

## 1 Introduction

Against a continuing global cancer burden (1), antibody-drug conjugates (ADCs) have become an established means of coupling antigen recognition to intracellular delivery of highly potent payloads. Their success, however, has not eliminated the central pharmacological problem of targeted delivery: tumour exposure must increase without proportionally increasing normal-tissue or payload-mediated toxicity. Antigen heterogeneity, incomplete internalisation, variable lysosomal processing, systemic release of payload and low-level target expression in normal tissues can all narrow this therapeutic window (2–4).

Bispecific antibody-drug conjugates (BsADCs) add a second antigen- or epitope-binding specificity to this already complex system. Dual-antigen formats may broaden coverage across heterogeneous tumour-cell populations, whereas biparatopic formats can alter receptor clustering and trafficking. Yet a second binding arm is useful only when it changes tumour selectivity, intracellular delivery or exposure sufficiently to offset the additional liabilities created by avidity, altered biodistribution, product complexity and payload toxicity (5–7). This distinction is now clinically testable rather than purely conceptual: in 2026, izalontamab brengitecan (iza-bren; BL-B01D1), an EGFR×HER3 BsADC, became the first BsADC to receive regulatory approval, first in recurrent or metastatic nasopharyngeal carcinoma and subsequently in recurrent or metastatic oesophageal squamous cell carcinoma (8, 9). The distinction between conventional single-target ADCs and bispecific formats is summarised in **Figure 1**.

**Figure 1:**
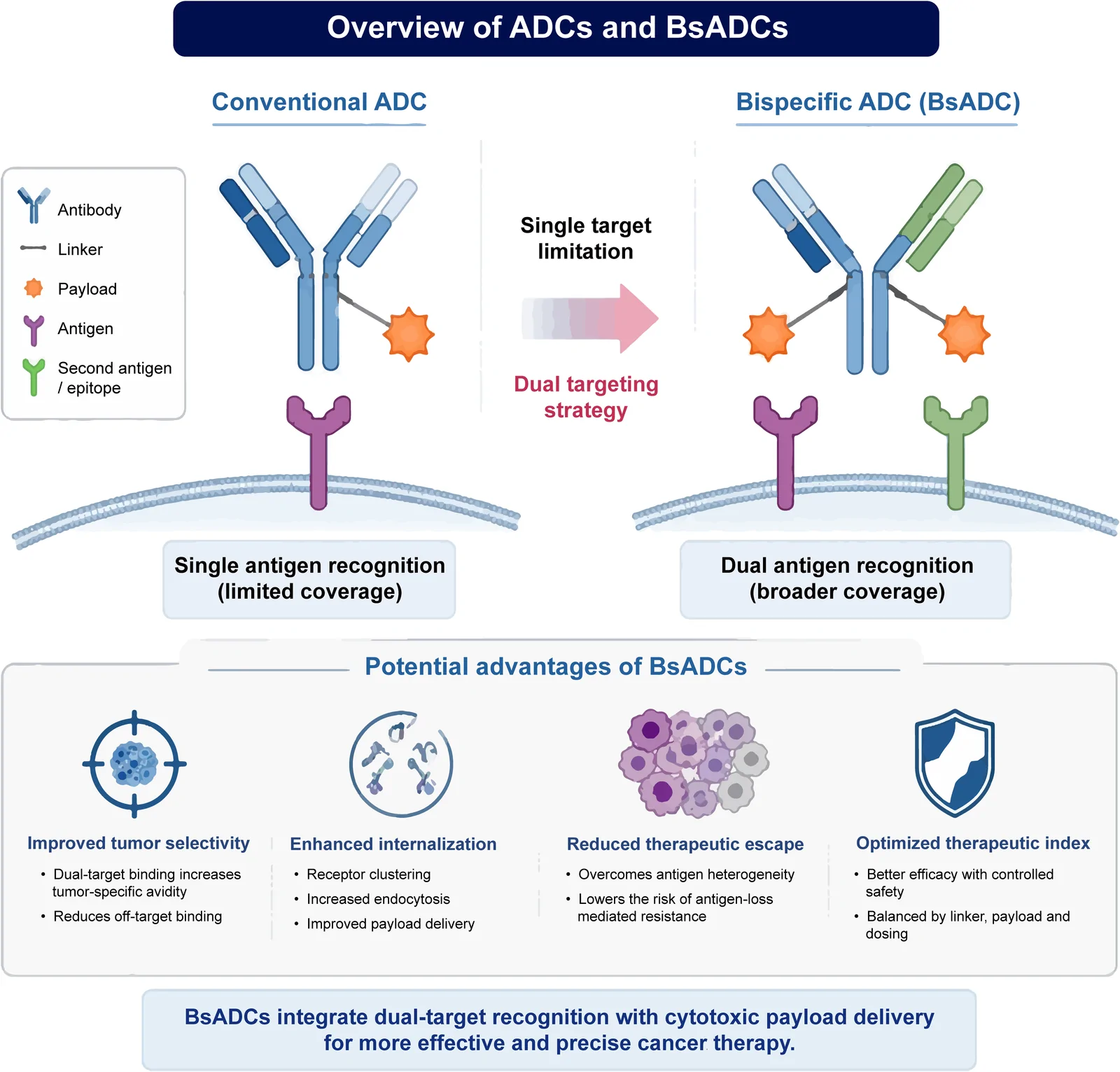
Overview of conventional antibody-drug conjugates and bispecific antibody-drug conjugates. Conventional antibody-drug conjugates (ADCs) recognize a single tumour-associated antigen, whereas bispecific antibody-drug conjugates (BsADCs) are designed to engage two distinct antigens or two epitopes on the same antigen. Depending on target co-expression, binding affinity and geometry, receptor trafficking, linker-payload design and dosing, bispecific recognition may broaden tumour coverage, enhance receptor clustering and internalisation, and reduce escape associated with loss of a single antigen. These potential advantages do not inherently guarantee improved selectivity or a wider therapeutic window and must be balanced against normal-tissue binding, altered biodistribution and payload-related toxicity. ADC, antibody-drug conjugate; BsADC, bispecific antibody-drug conjugate.

This review therefore asks two linked questions: which features of bispecific recognition actually widen the therapeutic window, and under what conditions do tumour-immune interactions enlarge or constrain that window? We organize the evidence around Target-Pair Biology and Co-Expression, Binding Geometry and Avidity, Internalisation and Lysosomal Trafficking, Linker-Payload Chemistry and Drug-to-Antibody Ratio, Intratumoural Distribution and Bystander Activity, and Biomarker-Guided Dosing and Patient Selection. Recent reviews have mapped BsADC components, antibody formats and development programmes (10); here, these architectures are examined through a linked therapeutic-window framework that connects design variables to tumour-immune modifiers and clinical translation. We then reassess resistance mechanisms, the tumour-immune microenvironment, immunogenic consequences of ADC payloads, immune-based combinations and the clinical pipeline, with evidence updated through 1 September 2026, and close by considering which preclinical measurements remain informative when development moves into human pharmacology.

## 2 From conventional ADC constraints to the BsADC therapeutic-window problem

### 2.1 Target heterogeneity, normal-tissue expression and internalisation

ADCs are modular therapeutics that combine the target selectivity of monoclonal antibodies with the cytotoxic activity of small-molecule payloads. A conventional ADC is composed of three essential elements: an antibody, a chemical linker and a cytotoxic payload (11). Its therapeutic performance depends not on a single component, but on the coordinated optimization of antigen selection, antibody properties, linker stability, payload potency and drug-to-antibody ratio (DAR).

A conventional ADC succeeds only when several conditions align: sufficient target density on malignant cells, limited normal-tissue accessibility, productive internalisation, stable circulation and efficient intracellular payload release. These requirements explain why antigen abundance alone is an incomplete predictor of efficacy. Targets with moderate expression but rapid internalisation and lysosomal trafficking may support effective delivery, whereas highly expressed targets can perform poorly if the ADC-antigen complex recycles or is misrouted (3, 11, 12).

Bispecificity can address two distinct failure modes. Dual-antigen BsADCs can increase the probability that at least one relevant target is present across heterogeneous tumour regions, while biparatopic ADCs can cross-link non-overlapping epitopes on the same receptor to promote clustering and internalisation. The same mechanisms can also increase risk: binding to either target may permit uptake in normal tissue, and high avidity may amplify exposure where one antigen is expressed at low but biologically relevant levels. Target-pair selection therefore requires co-expression and normal-tissue mapping rather than two independent single-antigen prevalence estimates (5–7).

The translational objective is not maximal binding but differential binding under clinically relevant antigen densities. For dual-antigen formats, the desired state is preferential retention where both targets coexist while preserving limited engagement of single-positive normal tissues. For biparatopic formats, the desired state is productive receptor clustering without excessive surface trapping or non-tumour uptake. These properties should be tested across heterogeneous models and, where possible, linked to quantitative target density, internalisation kinetics and human tissue cross-reactivity.

### 2.2 Payload exposure, bystander effect and systemic toxicity

After internalisation, BsADCs remain subject to the same exposure constraints as other ADCs. Linker stability, intracellular cleavage, payload potency, membrane permeability and DAR determine how much active drug reaches tumour cells and how much reaches non-malignant compartments. Higher DAR or a highly permeable payload may increase tumour-cell killing, but can also accelerate clearance, increase aggregation or amplify haematological, hepatic and other payload-related toxicities (4, 12–14).

Bystander activity is particularly relevant when dual targeting is intended to compensate for antigen heterogeneity. Cleavable linkers and membrane-permeable payloads can extend cytotoxicity to adjacent antigen-low or antigen-negative cells, but the same diffusion undermines the selectivity gained through antibody binding when payload reaches neighbouring normal cells. Its value therefore depends on spatial antigen distribution, tumour architecture and local payload exposure rather than on membrane permeability alone (15, 16).

The canonical sequence of antigen binding, endocytosis, endosomal-lysosomal trafficking and payload release remains useful for interpreting failure points, but a BsADC adds an upstream geometry problem: which receptor or receptor pair drives internalisation, at what antigen density, and with what consequences for systemic exposure. Productive internalisation must therefore be interpreted together with pharmacokinetics, tumour-immune context and toxicity rather than treated as a stand-alone efficacy endpoint.

### 2.3 A BsADC-specific therapeutic-window framework

Resistance can emerge at several points after target binding, including altered internalisation or lysosomal processing, drug efflux, changes in payload targets and compensatory survival signalling (17). For BsADCs, these failure modes are coupled to the additional variables introduced by dual recognition: a gain in tumour coverage or receptor handling has value only if payload delivery and systemic exposure remain compatible with a usable dose.

The relevant comparator is therefore an optimized monospecific ADC built with a clinically competitive linker-payload system, not bispecificity in isolation. Incremental value should be visible in tumour coverage, delivery, exposure or biomarker-defined activity without being offset by added toxicity or dosing burden. The conventional ADC failure modes summarised in **Figure 2** remain relevant, but bispecific design couples them to the additional constraints examined through the six determinants in Section 3.

**Figure 2:**
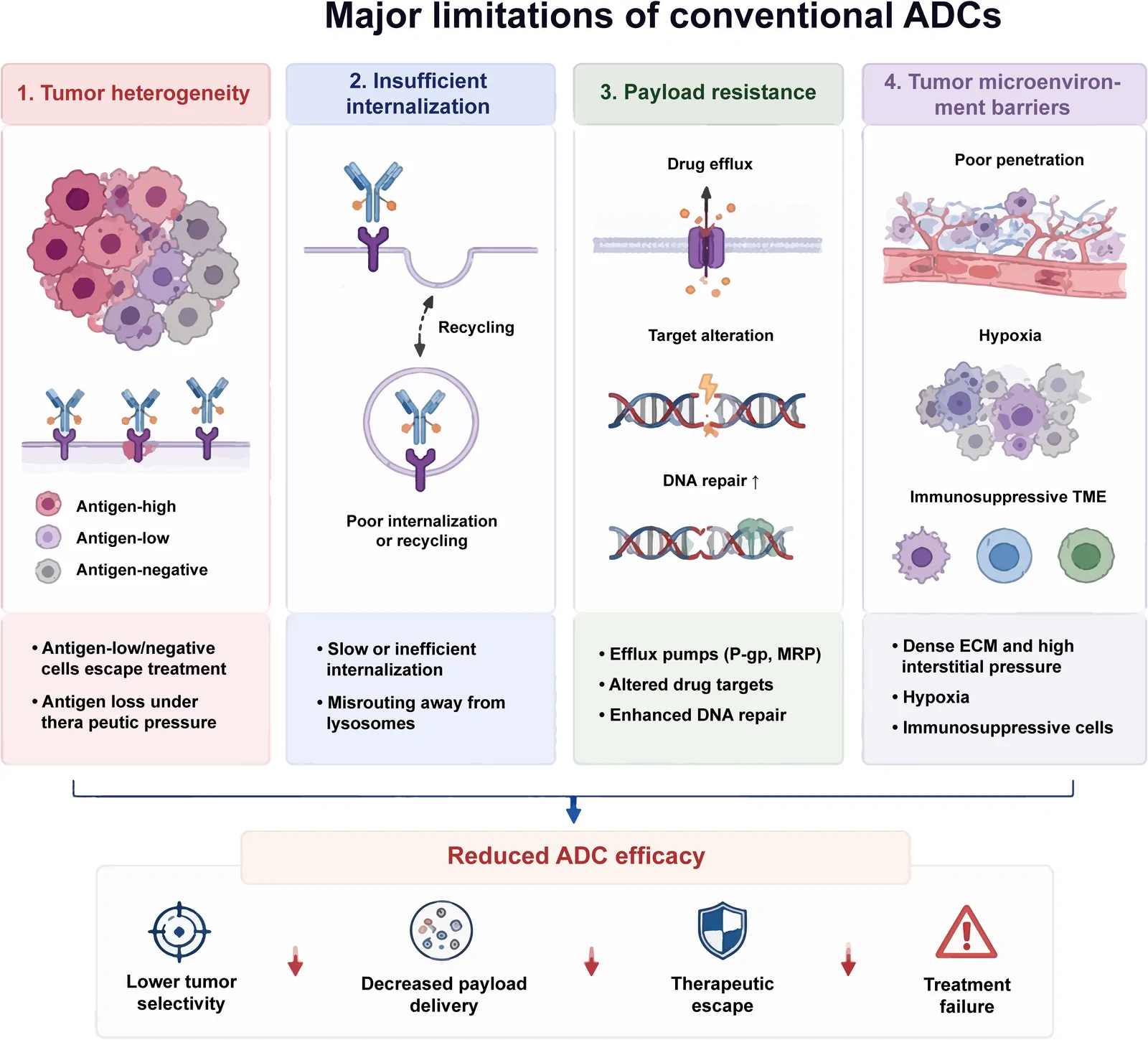
Major limitations of conventional antibody-drug conjugates. Antibody-drug conjugate activity may be limited by heterogeneous or reduced antigen expression, inefficient internalisation or recycling away from lysosomal compartments, payload resistance through drug efflux, altered payload targets or enhanced DNA repair, and tumour-microenvironmental barriers including poor penetration, dense extracellular matrix, hypoxia and immunosuppressive immune-cell populations. These mechanisms can reduce tumour-selective payload delivery and contribute to therapeutic escape or treatment failure. Many of these failure modes remain relevant to BsADCs and provide the biological rationale for selected bispecific design strategies. ADC, antibody-drug conjugate; BsADC, bispecific antibody-drug conjugate; DNA, deoxyribonucleic acid; ECM, extracellular matrix; MRP, multidrug resistance-associated protein; P-gp, P-glycoprotein; TME, tumour microenvironment.

## 3 Six therapeutic-window determinants of bispecific ADCs

Bispecific ADC formats are architectures through which linked design variables operate, not therapeutic categories with fixed advantages. Dual-antigen, biparatopic and multispecific constructs can alter tumour recognition, receptor handling or payload delivery only insofar as those changes remain compatible with product pharmacology and human dosing (5, 6). The six determinants below provide a sequential framework for evaluating the therapeutic window (**Table 1**).

**Table 1:** BsADC-specific therapeutic-window determinants and translational readouts.

Design determinant	Intended advantage	Principal liability	Translational readout
Target-Pair Biology and Co-Expression	Conditional tumour retention across heterogeneity	Single-positive normal-tissue uptake	Paired/spatial expression; normal-tissue map; response/safety threshold exploration
Binding Geometry and Avidity	Conditional clustering or tumour retention	Surface trapping or non-tumour avidity	Density-dependent binding/clustering; tissue cross-reactivity
Internalisation and Lysosomal Trafficking	Productive intracellular delivery	Recycling, misrouting or target variability	Endocytosis/recycling; lysosomal routing; intracellular payload
Linker-Payload Chemistry and Drug-to-Antibody Ratio	Controlled payload carriage and release	Instability, free payload, aggregation or rapid clearance	Intact ADC/total antibody/free-payload PK; DAR stability; exposure-toxicity
Intratumoural Distribution and Bystander Activity	Coverage of heterogeneous tumour regions	Loss of cellular selectivity; adjacent-tissue exposure	Tumour uptake/spatial exposure; target-density response; neighbouring-cell kill
Biomarker-Guided Dosing and Patient Selection	Maintain benefit at a usable exposure	Suboptimal dose intensity or unselected population	Target thresholds; PK/PD; exposure-response/toxicity; early toxicity

ADC, antibody-drug conjugate; DAR, drug-to-antibody ratio; PK/PD, pharmacokinetics/pharmacodynamics.

### 3.1 Target-pair biology and co-expression

A second target is useful when its joint distribution with the first creates a tumour-favouring binding opportunity, not simply because both antigens are individually prevalent. In an iza-bren study in oesophageal squamous cell carcinoma, pretreatment samples showed frequent EGFR/HER3 co-expression, yet neither single-marker H-score showed a clear relationship with response (18). Cell-level digital pathology has also mapped EGFR/HER3 co-expression across tumour and normal tissues (19), resolving dual-positive and single-positive compartments within the same specimen. Paired and spatial mapping is more informative than two separate prevalence estimates because it captures both tumour coverage and potential normal-tissue uptake, although the current evidence stops short of a validated selection threshold. The relevant target-pair readout is product- and indication-specific co-expression linked to response and safety.

### 3.2 Binding geometry and avidity

Once a target or epitope pair is chosen, geometry determines how the antibody engages receptors across realistic antigen densities. The aim is not maximal affinity or valency, but receptor organisation that favours tumour retention or productive complex formation without disproportionate binding to single-positive or normal tissues. Biparatopic HER2 engagement provides the clearest mechanistic example: binding two non-overlapping epitopes promoted receptor clustering rather than simply increasing nominal HER2 affinity (20). A biparatopic MET design similarly used dual-epitope engagement to alter receptor organisation (21). Avidity is most informative when profiled across antigen-density gradients and tissue cross-reactivity, where it can be interpreted as a conditional property of the target context rather than an intrinsic measure of quality (**Figure 3**).

**Figure 3:**
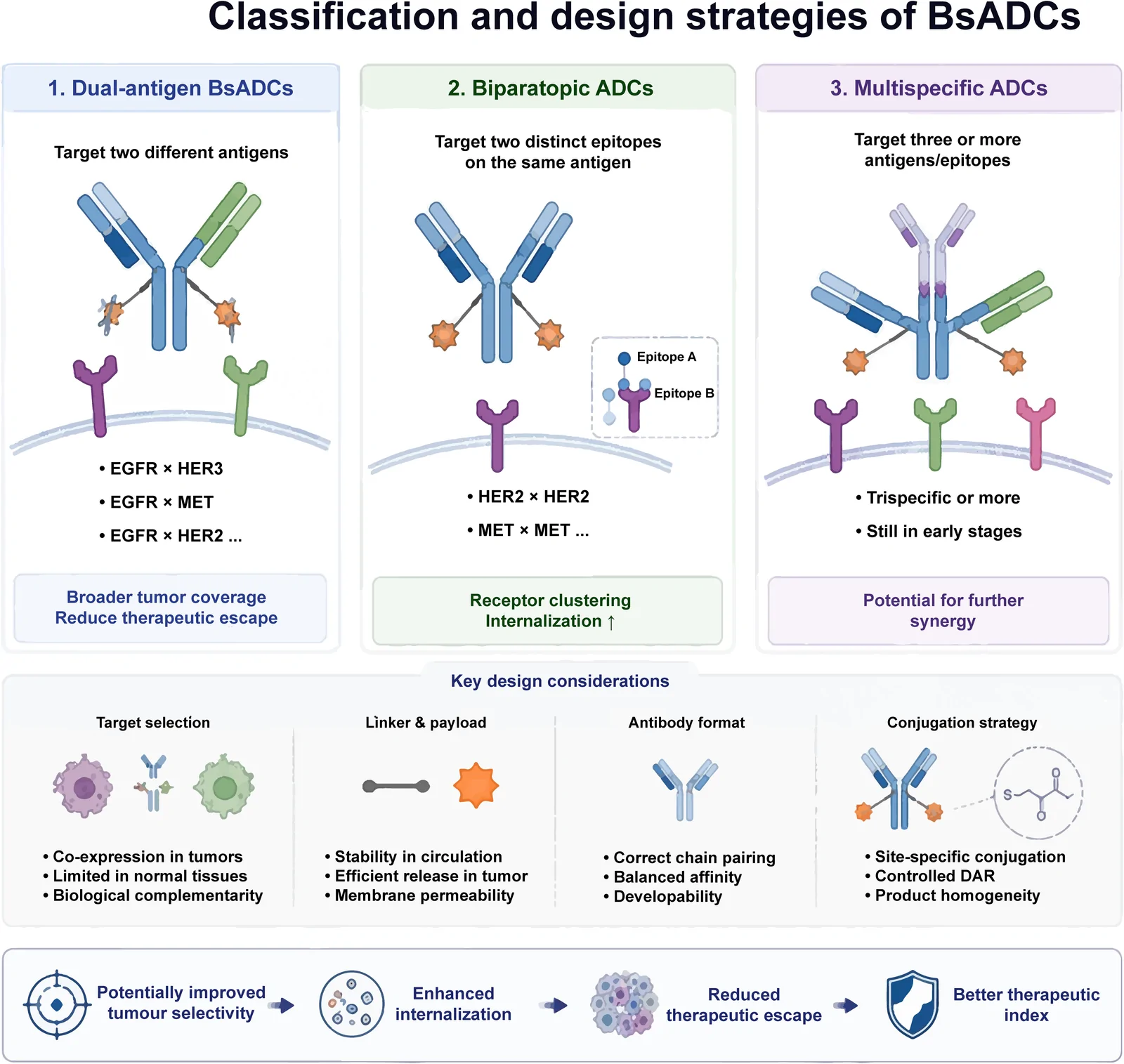
Classification and design strategies of bispecific antibody-drug conjugates. Bispecific antibody-drug conjugates (BsADCs) can be broadly classified into dual-antigen formats that recognize two different tumour-associated antigens, biparatopic formats that engage two non-overlapping epitopes on the same antigen, and emerging multispecific formats that recognize three or more antigens or epitopes. Dual-antigen recognition may broaden tumour coverage or support conditional avidity, whereas biparatopic binding can promote receptor clustering, internalisation and lysosomal trafficking. Rational development requires coordinated optimization of target co-expression, antibody geometry and affinity, linker-payload properties, drug-to-antibody ratio, conjugation strategy, manufacturability and tumour distribution. The resulting effects on tumour selectivity, therapeutic escape and therapeutic window remain context dependent and require experimental and clinical validation. BsADC, bispecific antibody-drug conjugate; DAR, drug-to-antibody ratio; EGFR, epidermal growth factor receptor; HER2, human epidermal growth factor receptor 2; HER3, human epidermal growth factor receptor 3; MET, MET receptor tyrosine kinase.

### 3.3 Internalisation and lysosomal trafficking

Geometry matters therapeutically only when receptor engagement produces useful intracellular delivery. Internalisation, recycling and lysosomal routing are distinct readouts because receptor uptake can still be followed by membrane recycling or non-productive intracellular routing. In the HER2 biparatopic system, receptor clustering was followed by robust internalisation, lysosomal trafficking and receptor degradation (20). REGN5093-M114 provides a second receptor context in which rapid internalisation, inefficient recycling and lysosomal routing were linked to ADC delivery (21). MEDI4276 retained the same broad receptor-handling logic but remained constrained by the exposure patients could tolerate (22). Together, these studies place the informative endpoint downstream of uptake itself: productive lysosomal delivery must be interpreted with intracellular payload exposure, the fraction of product that reaches tumour cells, and the systemic dose that can actually be sustained.

### 3.4 Linker-payload chemistry and drug-to-antibody ratio

Bispecific recognition cannot compensate for unfavourable product pharmacology. Linker stability, payload permeability and potency, DAR and hydrophobicity shape circulating exposure, clearance and off-tumour payload availability. High drug loading is not an objective in itself because greater payload carriage can increase hydrophobicity, aggregation or premature clearance. In conventional ADC systems, increasing hydrophobicity at high DAR accelerated clearance, whereas a more hydrophilic drug-linker improved pharmacokinetics and therapeutic index (14). In biparatopic products, site-specific conjugation can improve homogeneity and make structure–activity relationships more interpretable, as illustrated by glycan-conjugated JSKN003 (23). The benefit lies in control of product properties rather than assuming that a conjugation method is intrinsically superior (24).

Human programmes can resolve these properties directly rather than infer them from administered dose. Studies of biparatopic and bispecific ADCs have separately measured total antibody, conjugated ADC and released or deconjugated payload species (18, 22). Multi-analyte pharmacokinetics distinguishes antibody exposure, loss of conjugated drug and free-payload exposure, helping to identify whether apparent dose escalation is actually increasing intact-product delivery or mainly increasing released payload. These measurements provide a direct test of whether linker, payload, DAR and conjugation architecture generate stable systemic pharmacology. Exposure–response and exposure–toxicity relationships remain product-specific; this subsection defines the pharmacological measurements, while dose selection from those measurements is addressed in Section 3.6.

### 3.5 Intratumoural distribution and bystander activity

Tumour localisation and bystander killing are related but separable properties. A BsADC may localise efficiently yet release little membrane-permeable payload, or it may generate substantial local payload spread after internalisation. REGN5093-M114 illustrates the first dimension: PET uptake of radiolabelled biparatopic MET antibody tracked with MET expression and antitumour activity in the tested models (21). Monospecific ADC studies separately show how tissue penetration and local payload permeability can be measured (15, 16). Distribution determines where intact product accumulates, whereas bystander activity determines how far released payload can extend killing beyond directly targeted cells. The required degree of local spread depends on target heterogeneity, the spatial proximity of target-low cells and the risk of extending payload exposure into adjacent normal tissue. Distribution and bystander activity are best measured independently rather than treated as a single proxy for tumour coverage.

### 3.6 Biomarker-guided dosing and patient selection

Biomarker development is most useful when the measured variable matches the product mechanism. In preclinical REGN5093-M114 studies, cell-surface MET expression was more informative for efficacy than MET gene copy number in the tested models (25), showing that a genomic proxy may be less useful than the surface substrate actually available for binding and delivery. Human iza-bren data provide a complementary boundary: EGFR and HER3 were frequently co-expressed in pretreatment samples, but neither individual H-score defined a clear response threshold (18). The contrast favours product-matched measurements of target quantity and spatial distribution over generic target positivity. It also separates biomarker usefulness from biomarker validation: a biologically relevant assay can guide development before a predictive threshold has been established for the specific product and indication.

Dose selection extends the same logic from tissue measurement to human exposure. In the first-in-human iza-bren programme, the recommended phase II dose integrated safety, tolerability, activity, pharmacokinetic and pharmacodynamic information rather than target expression alone (26); subsequent multi-analyte measurements linked dose to antibody, intact ADC and released-payload exposure (18). These data illustrate why patient selection and dosing cannot be separated once the product reaches the clinic: the target may define where a BsADC can act, but exposure and toxicity determine whether that opportunity can be maintained at an effective dose intensity. Biomarker-guided development is most useful when target biology can be connected to an exposure that remains clinically usable, rather than when a universal BsADC threshold is assumed.

## 4 Mechanisms of resistance and tumour-immune constraints in ADCs and BsADCs

### 4.1 Framework for classifying resistance mechanisms

ADC and BsADC resistance can arise at sequential steps spanning target recognition, internalisation, lysosomal processing, payload release, payload sensitivity and adaptive signalling (2–4, 17, 27). Bispecificity can plausibly mitigate selected target- or trafficking-level failures, but it does not remove downstream payload resistance or microenvironmental restriction. Accordingly, **Table 2** distinguishes mechanistic mitigation from direct clinical proof; most proposed BsADC rescue strategies remain context dependent.

**Table 2:** Resistance mechanisms, potential BsADC mitigation and evidence boundaries.

Resistance level	Principal mechanism	Potential BsADC mitigation and evidence boundary
Target-level	Antigen loss, epitope alteration, spatial/temporal heterogeneity	Dual-antigen or dual-epitope recognition may broaden coverage; clinical proof that this prevents acquired escape is limited.
Internalisation/trafficking	Slow uptake, recycling, misrouting away from lysosomes	Biparatopic or bispecific binding may promote clustering and lysosomal delivery; MEDI4276 shows that improved internalisation can still coexist with an inadequate therapeutic window.
Lysosomal	Altered acidification, protease activity or processing	Linker chemistry may improve release, but bispecificity itself does not correct lysosomal dysfunction.
Payload-level	Drug efflux, altered payload target, enhanced DNA repair, insufficient intracellular concentration	Alternative or dual payloads may reduce selected resistance mechanisms; added payload diversity also increases toxicity and PK complexity.
Signalling-pathway	PI3K/AKT, MAPK, HER3, MET or other bypass signalling	Target pairing or rational combinations may suppress selected bypass pathways; benefit requires prospective clinical validation.
Tumour microenvironment	Poor perfusion, dense ECM, high interstitial pressure, hypoxia and immunosuppressive niches	Bispecific recognition does not inherently improve macromolecular penetration; TME-directed combinations remain hypothesis-driven and may add systemic toxicity.

ADC, antibody-drug conjugate; BsADC, bispecific antibody-drug conjugate; ECM, extracellular matrix; PK, pharmacokinetics; TME, tumour microenvironment.

Resistance at the target level is most commonly driven by reduced antigen expression, antigen loss, or structural alteration of the target epitope, all of which can impair ADC binding. Solid tumours frequently exhibit substantial antigen heterogeneity both within primary lesions and across metastatic sites; under therapeutic pressure, antigen-high clones may be depleted, whereas antigen-low or antigen-negative subclones may become enriched (3, 27). In DESTINY-Breast04, which enrolled 557 patients with HER2-low advanced breast cancer, trastuzumab deruxtecan (T-DXd) significantly improved progression-free and overall survival compared with standard chemotherapy. These findings suggest that membrane-permeable payloads and bystander killing may partly overcome antigen heterogeneity, although interstitial lung disease and myelosuppression remain clinically relevant safety concerns (28). In this context, BsADCs may broaden target coverage by recognising two antigens or distinct epitopes, potentially reducing the likelihood of escape mediated by loss of a single target (5).

Defective internalisation represents another important mechanism of resistance. Even when surface antigen expression is preserved, therapeutic efficacy may be compromised if the ADC-antigen complex is internalised inefficiently, recycled back to the plasma membrane, or misdirected away from the endosomal-lysosomal pathway (2, 3). Dual-epitope ADCs and BsADCs may promote receptor crosslinking and clustering, thereby enhancing internalisation and lysosomal delivery. In a phase 1 study reported by Jänne and colleagues, HER3-DXd showed antitumour activity in patients with EGFR-mutated non-small cell lung cancer after resistance to EGFR inhibitors, supporting the concept that payload delivery through compensatory receptors may help address resistance. However, this evidence remains early, and longer follow-up is required to define the durability of benefit and safety profile (29).

Resistance at the lysosomal level is closely linked to inefficient payload release. Most ADCs require lysosomal acidification, proteolytic degradation, or linker cleavage to liberate the active cytotoxic payload. Impaired lysosomal acidification, reduced protease activity, altered lysosomal trafficking, or remodelling of autophagy-lysosome pathways may therefore limit payload release and cytosolic access (2, 4). Non-cleavable linkers depend on complete antibody degradation, whereas cleavable linkers must balance systemic stability with selective cleavage in tumour cells. The clinical superiority of T-DXd over trastuzumab emtansine (T-DM1) in HER2-positive breast cancer may be related to its higher drug-to-antibody ratio, cleavable tetrapeptide linker, and topoisomerase I inhibitor payload (30).

Payload-level resistance may arise from reduced sensitivity to the cytotoxic agent or insufficient intracellular drug concentration. Payloads such as MMAE, DM1, SN-38, and DXd act through microtubule disruption or DNA damage-related mechanisms; alterations in the payload target, enhanced DNA repair capacity, or upregulation of ATP-binding cassette transporters such as P-gp, MRP, and BCRP can attenuate cytotoxic activity (2, 4). Microtubule-inhibitor payloads may be particularly vulnerable to drug efflux, whereas some topoisomerase I inhibitor payloads have greater membrane permeability and can mediate bystander killing, potentially improving activity in antigen-heterogeneous tumours (4, 31). As ADCs such as enfortumab vedotin and sacituzumab govitecan become more widely used, the possibility of cross-resistance between agents sharing similar payloads or cytotoxic pathways warrants increasing attention (31, 32).

Adaptive signalling provides a further layer of resistance. Even when ADC binding, internalisation, and payload release occur successfully, tumour cells may maintain survival through activation of PI3K/AKT, MAPK, HER3, MET, or other bypass pathways, or through epithelial-mesenchymal transition, anti-apoptotic signalling, and enhanced DNA damage response (27, 29). BsADCs may reduce pathway compensation through dual-target engagement, and combinations with pathway inhibitors or immunotherapy have a strong biological rationale. Nevertheless, the clinical benefit, optimal sequencing, and safety of such combinations require prospective evaluation, particularly given the potential for overlapping toxicities (5, 27, 30). A structured overview of resistance mechanisms and potential BsADC-based mitigation strategies is provided in **Table 2**.

### 4.2 Tumour-immune microenvironment as a determinant of BsADC activity

The tumour microenvironment can restrict BsADC activity before any target-pair advantage is realized. Abnormal vasculature, high interstitial pressure, dense extracellular matrix and hypoxia produce uneven macromolecular exposure, while tumour-associated macrophages, myeloid-derived suppressor cells, regulatory T cells and dysfunctional or exhausted T cells can limit the immune consequences of tumour-cell killing (3, 27). Dual targeting may improve recognition of malignant cells, but it does not by itself correct stromal exclusion, defective antigen presentation or suppressive myeloid signalling.

ADC-mediated tumour-cell death can nevertheless be immunologically active, and this effect depends strongly on the payload and cellular context. In immunocompetent models, trastuzumab deruxtecan increased dendritic-cell activation, tumour-infiltrating CD8+ T cells, MHC class I and PD-L1 expression, and its activity was enhanced by PD-1 blockade (33). Pyrrolobenzodiazepine- and tubulysin-bearing ADCs have likewise produced immunogenic cell-death features, CD8-dependent antitumour effects and synergy with several immunotherapies (34). More recently, HER3-DXd and its DXd payload were shown to induce calreticulin exposure, ATP release and HMGB1 release, supporting the view that topoisomerase I inhibitor payloads can contribute to immune priming in selected settings (35). These observations are mechanistically relevant to BsADCs but are not evidence that bispecific recognition itself is immunogenic.

The antibody component adds another immunological layer. Fc-dependent effector functions may contribute to antibody-dependent cellular cytotoxicity or phagocytosis, whereas Fcγ receptor-mediated uptake by target-negative myeloid cells can also increase off-target payload exposure. Experimental work with ADC aggregates has shown enhanced Fcγ receptor-dependent internalisation and cytotoxicity in target-negative Fcγ receptor-expressing cells, illustrating how product quality and Fc biology can become safety determinants as well as immune-effectors (36). For BsADCs, altered avidity, geometry and biodistribution may change these interactions further, making Fc design, aggregation control and human tissue cross-reactivity relevant to both efficacy and toxicity.

Tumour-immune context modifies the therapeutic window rather than defining an intrinsic property of BsADCs. Paired target distribution can be interpreted alongside antigen-presentation competence, myeloid suppression and treatment-induced changes in dendritic cells, CD8+ T cells and checkpoint ligands when tissue is available. Because direct immune-mechanistic evidence remains largely derived from monospecific ADCs, its role here is to indicate when payload and Fc biology may enlarge or constrain the window created by bispecific targeting.

## 5 Clinical translation, immunotherapy integration and regulatory validation of bispecific ADCs

### 5.1 Iza-bren: from first-in-human activity to regulatory approval

The clinical landscape changed materially in 2026. On 22 June 2026, China’s NMPA approved izalontamab brengitecan (iza-bren; BL-B01D1) for recurrent or metastatic nasopharyngeal carcinoma after progression following prior platinum-based chemotherapy and PD-1/PD-L1 inhibitor therapy; the sponsor described this as the first regulatory approval of any bispecific ADC. On 17 July 2026, a second NMPA approval followed for adult patients with recurrent or metastatic oesophageal squamous cell carcinoma after progression following platinum-based chemotherapy and PD-1/PD-L1 inhibition (8, 9). These approvals invalidate the earlier view of BsADCs as an entirely pre-approval modality and provide a regulatory anchor for evaluating the rest of the field.

The nasopharyngeal carcinoma approval was supported by the randomized phase III BL-B01D1–303 study. Iza-bren improved confirmed objective response, duration of response and progression-free survival versus physician’s-choice chemotherapy; the peer-reviewed phase III report and subsequent regulatory decision establish this as the most mature human evidence for the modality (8, 37). In PANKU-Esophagus01, iza-bren improved median overall survival (9.8 versus 7.2 months; HR 0.64) and progression-free survival (4.2 versus 2.0 months; HR 0.50) compared with chemotherapy, supporting the second approval (9, 38).

The phase III PANKU-Breast02 study further showed that the regulatory success is not confined to one tumour type. In previously treated unresectable locally advanced or metastatic triple-negative breast cancer, iza-bren improved median progression-free survival (8.5 versus 3.1 months), median overall survival (15.9 versus 12.5 months) and confirmed objective response rate (51.7% versus 20.5%) versus physician’s-choice chemotherapy (39). Outside mainland China, iza-bren is being developed under the BMS–SystImmune global collaboration; the U.S. FDA Breakthrough Therapy Designation applies to previously treated EGFR-mutant NSCLC, not NPC (40). As of 1 September 2026, a dedicated ex-China regulatory pathway for NPC had not been publicly disclosed (41, 42).

The translational lesson from iza-bren is not simply that dual targeting works. Haematological toxicity remains prominent, ILD requires active surveillance, and efficacy varies across tumour types and treatment settings (18, 26, 37, 43–45). Its development instead supports a more specific interpretation: EGFR×HER3 co-targeting can produce a workable therapeutic window when paired with an appropriate topoisomerase I payload, schedule, dose modification strategy and clinical population. The remaining question is which of those elements are generalizable to other BsADCs. An overview of the clinical development status and therapeutic-window lessons of selected BsADC and biparatopic ADC programmes is provided in **Table 3**.

**Table 3:** Human clinical evidence and development status of selected BsADC and biparatopic ADC programmes as of 1 September 2026.

Programme/target	Construct/payload	Clinical status/key evidence (1 Sep 2026)	Therapeutic-window lesson
Iza-bren (BL-B01D1) EGFR×HER3	Bispecific ADC; Topo1 payload	China approvals in R/M NPC and R/M ESCC; randomized phase III benefit in NPC, ESCC and TNBC; U.S. BTD for EGFR-mutant NSCLC	Workable window reflects complete-product pharmacology plus dose/schedule; haematologic toxicity and ILD constrain exposure.
JSKN003 HER2×HER2	Biparatopic HER2; site-specific glycan conjugation; Topo1 payload	Phase III/registrational; multiple studies ongoing	Tests whether conjugation control and biparatopic delivery remain compatible with safety.
JSKN016 TROP2×HER3	Bispecific ADC; Topo1 payload	Phase III TNBC after early clinical activity	Needs a competitive comparator to resolve added target-pair value.
EGFR×MET programmes AZD9592/DM005/HLX48	Bispecific EGFR×MET ADCs; Topo1-class payloads for AZD9592/HLX48; DM005 bispecific ADC	AZD9592 phase I recruitment complete; DM005 phase I with 2026 FIH data; HLX48 phase I, first patient dosed June 2026	Distinct product platforms test co-expression, normal-tissue exposure and dose without assuming class equivalence.
IMGN151 FRα×FRα	Biparatopic FRα ADC; maytansinoid derivative	Phase II; recruiting (NCT07024784)	Heterogeneous target coverage must remain compatible with ocular/systemic tolerability.
MEDI4276 HER2×HER2	Tetravalent biparatopic HER2; tubulysin	Phase I completed; clinical responses with a narrow tolerated dose range	Receptor handling did not by itself define a broad usable exposure range.
Zanidatamab zovodotin (ZW49) HER2 biparatopic	Biparatopic HER2 ADC; auristatin	Development discontinued in 2024 after phase I activity	Clinical activity alone does not establish a sustainable product profile.
M1231 MUC1×EGFR	Bispecific ADC; hemiasterlin-related payload	Phase I completed; NCT04695847; 23 participants	Development has not progressed beyond the completed early-phase study.
REGN5093-M114 MET×MET	Biparatopic MET ADC; maytansinoid	Clinical study terminated; NCT04982224	Target-density, trafficking and uptake readouts require confirmation at human exposure.
JSKN021 EGFR×HER3	Bispecific dual-cytotoxic-payload ADC; Topo1 inhibitor + MMAE	Phase I; first patient dosed June 2026	Tests payload diversification with added PK, toxicity and manufacturing complexity.

ADC, antibody-drug conjugate; BTD, Breakthrough Therapy Designation; ESCC, oesophageal squamous cell carcinoma; FIH, first-in-human; ILD, interstitial lung disease; MMAE, monomethyl auristatin E; NPC, nasopharyngeal carcinoma; R/M, recurrent or metastatic; TNBC, triple-negative breast cancer; Topo1, topoisomerase I.

### 5.2 Pipeline lessons beyond iza-bren

Several active programmes now test distinct solutions to the therapeutic-window problem. JSKN003 has reached phase III/registrational development with a site-specific glycan-conjugated HER2 biparatopic design, while JSKN016 has entered phase III development in triple-negative breast cancer using TROP2×HER3 recognition (46, 47). IMGN151 remains in phase II development as a biparatopic FRα ADC (48). EGFR×c-MET programmes AZD9592, DM005 and HLX48 remain in phase I, providing opportunities to determine whether dual-receptor recognition improves tumour delivery without reproducing EGFR- or MET-associated normal-tissue liability (49–51).

Discontinued or terminated programmes are equally informative. MEDI4276 linked enhanced HER2 internalisation to a narrow tolerated dose range, demonstrating that receptor trafficking can improve while the overall therapeutic window worsens (22). ZW49 was discontinued after clinical development despite earlier phase I activity (52), while the REGN5093-M114 clinical study was terminated (53).

The pipeline is also beginning to test more complex product architectures. JSKN021 combines EGFR×HER3 recognition with two cytotoxic payloads and entered phase I in 2026 (54). Such designs are relevant only if added complexity produces a measurable gain in exposure-response or resistance control. The decisive translational evidence is therefore human PK/PD, dose intensity, discontinuation, clinically meaningful efficacy and biomarker-defined benefit rather than novelty of format.

### 5.3 BsADCs and immunotherapy: mechanistic rationale and clinical evidence

The rationale for combining an ADC with checkpoint blockade is strongest when payload-mediated tumour-cell death creates an immune-priming event rather than simply adding parallel cytotoxicity. Depending on the payload and cellular context, DNA damage or other treatment stress can produce immunogenic cell-death features such as calreticulin exposure and ATP or HMGB1 release, facilitating dendritic-cell uptake and maturation, antigen presentation and CD8+ T-cell priming. Treatment-induced interferon signalling and checkpoint-ligand expression can then create a setting in which PD-1/PD-L1 blockade sustains or restores the newly engaged T-cell response. T-DXd, pyrrolobenzodiazepine- or tubulysin-bearing ADCs, and HER3-DXd support different steps in this sequence (33–35). The sequence is not universal: ineffective antigen presentation or a strongly suppressive tumour microenvironment can interrupt it before checkpoint blockade becomes useful.

Clinical evidence shows that ADC–checkpoint combinations can improve outcomes, although the strongest data remain outside the BsADC field. In EV-302, enfortumab vedotin plus pembrolizumab improved progression-free and overall survival over platinum-based chemotherapy in untreated advanced urothelial carcinoma (55), and activity has also been reported with disitamab vedotin plus toripalimab in HER2-expressing advanced tumours (56). These studies establish clinical benefit for the tested combinations but do not identify bispecificity as the source of synergy.

For BsADCs, attribution becomes the key clinical issue. Combination trials can distinguish the contributions of target pairing, payload biology and checkpoint modulation by relating product and regimen exposure to efficacy and toxicity and by using baseline or on-treatment tissue and blood to track antigen presentation, T-cell recruitment and suppressive myeloid states. Marrow suppression, lymphopenia, interstitial lung disease and immune-related adverse events remain part of the same therapeutic-window calculation because each can reduce dose intensity. Immune modulation adds value when durable benefit is maintained at a regimen exposure patients can sustain.

## 6 Preclinical-to-clinical translation and next-generation development

### 6.1 Preclinical tools and translational readouts

No single preclinical model can test the therapeutic window as a whole. Each determinant is better matched to a specific readout: paired and spatial pathology with normal-tissue mapping for target-pair biology; antigen-density binding and clustering for geometry; endocytosis, recycling and lysosomal assays for trafficking; intact ADC, total antibody, free payload and DAR stability for product pharmacology; quantitative tumour and normal-organ localisation for distribution; and target, exposure and early-toxicity relationships for dose and biomarker development. Human tissue assays, CDX/PDX models and immune-competent systems answer complementary questions rather than form a universal hierarchy.

Assayability and predictiveness are not the same property. Human tissue can establish paired or spatial target distribution; tumour models can test target-dependent delivery and exposure; immune-competent systems are needed when immune context is part of the claim. Mechanistic confirmation becomes translationally informative only when the same variable—target, localisation, exposure or toxicity—remains measurable as development moves into human tissue or pharmacology. Fit-for-purpose continuity, rather than the number of models used, is the more useful standard.

### 6.2 Immunologically informed and dual-payload development

Dual-payload development encompasses two distinct design problems. One is cytotoxic-cytotoxic diversification, in which two payloads are intended to broaden the range of intracellular vulnerabilities or reduce dependence on a single resistance mechanism. JSKN021, for example, combines EGFR×HER3 recognition with two cytotoxic payloads and has entered phase I development (54). At this stage, the architecture demonstrates clinical testability rather than proven resistance control. Any gain must be weighed against added DAR heterogeneity, release kinetics, pharmacokinetic complexity and overlapping toxicity, because two active payloads can narrow rather than widen the exposure range if they cannot be dosed together effectively.

A different strategy couples tumour targeting with an explicitly immune-active payload. Antibody conjugates carrying TLR or STING agonists can concentrate innate immune stimulation at antigen-defined sites and activate myeloid programmes in preclinical models (57–59). These studies are mechanistic precedents for targeted immune-agonist delivery, not direct evidence for cytotoxic–immunostimulatory dual-payload BsADCs. For a BsADC, added value would need to come from improved localisation, cellular delivery or safety relative to a competitive monospecific construct; immune activation alone does not establish incremental bispecific value. Relevant readouts include local innate activation, systemic cytokine exposure and the durability of downstream immune changes.

For either architecture, product quality remains part of the biological test. Site-specific conjugation, controlled DAR, payload ratio, aggregation control and batch consistency influence exposure and make payload-specific pharmacokinetics interpretable (24, 36). Cytotoxic–cytotoxic designs test resistance coverage; cytotoxic–immunostimulatory designs test whether local immune priming can be added without sacrificing the therapeutic window. The additional complexity earns its place when those readouts reveal an advantage that a simpler construct cannot provide.

### 6.3 Comparative evidence and clinical priorities

Comparative evidence is most informative when the comparator is pharmacologically competitive. The T-DXd/T-DM1 experience illustrates this principle. Before randomized superiority was known, preclinical T-DXd studies had already differentiated the product through its linker–payload architecture, high DAR, activity in T-DM1-insensitive or lower/heterogeneous-HER2 models and local bystander killing (15, 60). Those product differences made a broader efficacy profile directionally plausible; they did not specify the later magnitude of benefit or the full human safety trade-off, including interstitial lung disease/pneumonitis (30). Preclinical differentiation is most useful as directional evidence that a product property may remain clinically relevant, not as a deterministic forecast.

Current BsADC programmes can be judged by the same standard. Iza-bren is informative because target-pair biology, complete-product pharmacology, dose selection and indication context remained compatible enough to produce randomized benefit, without isolating bispecificity as the causal feature. MEDI4276 shows the complementary boundary: strong receptor-handling design and measurable analyte pharmacokinetics can coexist with a narrow-tolerated exposure range (22). A clinically persuasive BsADC must preserve the incremental value of its second specificity after comparison with an optimized monospecific ADC and after human exposure, safety, dose intensity and biomarker selection are taken into account. Embedding these measurements during dose development distinguishes an active format from a product whose therapeutic-window advantage remains visible in patients.

## 7 Conclusions

Bispecific antibody-drug conjugates have moved from an experimental design concept to a clinically validated modality, but their success should not be interpreted as evidence that dual recognition is intrinsically superior to optimized monospecific ADCs. The therapeutic window remains the decisive endpoint and reflects the integrated effects of target-pair distribution, binding geometry, intracellular trafficking, linker-payload properties, tumour penetration, dose and patient selection. Immune biology adds a second layer to this framework. Selected ADC payloads can induce immunogenic cell death, alter dendritic-cell and T-cell activity, and create a rationale for checkpoint blockade, while Fcγ receptor-dependent uptake and suppressive tumour-immune niches may also increase off-target exposure or blunt durable immunity. At present, however, most of this immunological evidence comes from monospecific ADCs; BsADC-specific immune benefit remains to be demonstrated directly. Future development should therefore combine target-pair and immune-contexture biomarkers with human PK/PD, exposure-toxicity relationships and informative comparators. The most compelling next-generation BsADCs will be those in which the second specificity produces a measurable gain in tumour-selective delivery or immune-permissive tumour control without sacrificing dose intensity, manufacturability or safety.
